# Parallelized shifted‐excitation Raman difference spectroscopy for fluorescence rejection in a temporary varying system

**DOI:** 10.1002/jbio.201960028

**Published:** 2019-08-28

**Authors:** Rintaro Shimada, Takashi Nakamura, Takeaki Ozawa

**Affiliations:** ^1^ Department of Chemistry, Graduate School of Science The University of Tokyo Tokyo Japan

**Keywords:** background correction, mechanical streak camera, multivariate analysis, Raman spectroscopy, SERDS

## Abstract

A fluorescence background is one of the common interference factors of the Raman spectroscopic analysis in the biology field. Shifted‐excitation Raman difference spectroscopy (SERDS), in which a slow (typically 1 Hz) modulation to excitation wavelength is coupled with a sequential acquisition of alternating shifted‐excitation spectra, has been used to separate Raman scattering from excitation‐shift insensitive background. This sequential method is susceptible to spectral change and thus is limited only to stable samples. We incorporated a fast laser modulation (200 Hz) and a mechanical streak camera into SERDS to effectively parallelize the SERDS measurement in a single exposure. The developed system expands the scope of SERDS to include temporary varying system. The proof of concept is demonstrated using highly fluorescent samples, including living algae. Quantitative performance in fluorescence rejection and the robustness of the method to the dynamic spectral change during the measurement are manifested.

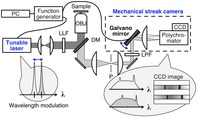

## Graphical abstract

We present parallelized shifted excitation Raman difference spectroscopy by the synchronized operation of a mechanical streak camera with a wavelength tunable light source. The parallel detection scheme facilitates the effective and accurate fluorescence rejection from a temporary varying system having faster dynamics than the spectral acquisition rate. Quantitative performance in fluorescence rejection and the robustness of the method to the dynamic spectral change during the measurement are manifested.

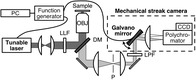

**ABBREVIATIONS**
CCD: charge‐coupled device
CW: continuous wave
deform.: deformation
MCR‐ALS: multivariate curve resolution‐alternate least squares
PCA: principal component analysis
P‐SERDS: parallelized shifted‐excitation Raman difference spectroscopy
str.: stretch

## 1 INTRODUCTION

In recent decades, the application of Raman spectroscopy to biological and medical studies has been expanded vastly, especially in the field of a living cell and tissue characterization 1, 2, 3, 4, 5, 6. One of the key driving forces of this recent expansion is the drastic advancement in the various chemometric data mining techniques for data decompositions, clustering and discriminant analyses 7, 8, 9, 10, 11. During the mining processes of Raman spectroscopic data set, the purity of the data is one of the decisive factors of the analytical performance. Inclusion of irrelevant signals such as a background to the input data set often disturbs the analysis and alters the output, which could lead to a vague or even wrong conclusion. Therefore, preclusion of the extraneous signal prior to the actual analysis is necessary for achieving a reliable outcome. In a biological application of Raman spectroscopy, fluorescence from either endogenous or exogenous biomolecules is usually the primary source of background. Although the magnitude of fluorescence can be greatly reduced by tuning the excitation laser to longer wavelength 12, the complete suppression of fluorescence from bio‐originated samples is extremely difficult even by 1064 nm excitation 13, 14. Hence, the development of techniques which facilitates the separation of Raman signal from the fluorescence background is deemed important.

Currently, the most frequently used branch of fluorescence eliminating techniques is an ad hoc baseline subtraction by polynomial curve fitting 12, 15. These methods attribute slowly varying spectral components to fluorescence based on the tendency of its broader bandwidth than Raman peaks. However, this assumption does not always hold for biological samples because ubiquitous biomolecules, such as proteins, DNAs and polysaccharides, are polymers, and these biopolymer Raman spectra exhibit broadened bandwidth and unclear baselines in the fingerprint region due to the presence of numerous vibrational modes and to their structural diversity. Moreover, the order of polynomial functions is often arbitrarily (or statistically) chosen so as to make the resultant spectrum have the flattest baseline. There is usually little physical justification in the process; thus, they are considered as less objective and expedient methods. Also, as we will show later in the result section, polynomial subtraction methods are not always compatible with chemometric analyses and may introduce an additional complication to the data set.

To overcome above issues, numbers of fluorescence rejection methods based on solid physical principles, such as temporal 16, 17 or spectral response differences 18, 19, 20, have been proposed. Of these methods, shifted‐excitation Raman difference spectroscopy (SERDS) 19 and its variants 20, 21, 22, 23, 24 are based on the principle that Raman and fluorescence spectra respond differently to small shifts in the excitation wavelength; Raman bands shift concertedly with the excitation light, whereas fluorescence does not. Previous studies have shown the effectiveness of the principle in extracting Raman signals from various fluorescent subjects, including cultured cells 23, human tissue 25 and tooth 24. Despite these successes, the application of SERDS methods to biological samples is still technically limited. The samples are constrained to be stable, giving a stationary response. This restriction exists because these methods consecutively record a series of Raman spectra excited by shifted wavelengths, and the samples are required to be unchanged during the whole set of measurement. In this sequential scheme, the spectral acquisition rate determines the wavelength switching frequency, which is typically in the order of seconds or longer. However, for many biological samples, especially the live ones, spectral changes may occur in much faster time scale by photobleaching 25, 26 and by the physical movement of components in and out of the laser focus.

In this study, we took a further step to expand the scope of the SERDS technique to include unstable, dynamic samples by effectively parallelizing the measurement. Herein, we first outline the principle of the developed parallelized SERDS (P‐SERDS). Then, proof of concept studies using stationary fluorescing artificial dye solution and dynamic living algae cells are reported. High‐quantitative performance in fluorescence rejection, as well as the robustness of the method to the dynamic spectral change during a signal accumulation, is manifested. Finally, we demonstrate the superior compatibility of the proposed method to multivariate chemometrics in comparison with the conventional fluorescence removal method using the polynomial baseline fitting.

## 2 PARALLELIZED SHIFTED‐EXCITATION RAMAN DIFFERENCE SPECTROSCOPY

Similar to other SERDS instruments, the P‐SERDS utilizes a wavelength tunable laser for fast wavelength switching. Parallelization of the measurement is realized by synchronization of fast laser tuning with a modulation to a mechanical streak camera. A mechanical streak camera is composed of a galvano mirror and a spectrograph, in which the galvano unit is used to control the height of signal light entering the spectrometer. Without invoking a detector readout, the device can designate and modulate the vertical position on the two‐dimensional detector pixels on which signal light is exposed. By driving the streak device to switch detection pixels repeatedly in conjunction with the laser tuning, it is possible to parallelly record multiple spectra excited by the different laser wavelengths in a single exposure.

The advantage of the present parallel configuration is the independence of the spectral acquisition rate and the modulation frequency. Long (seconds to minutes) exposures for weak Raman scatterers can be performed, while employing orders of magnitude faster wavelength switching frequency (up to 200 Hz). Owing to this fast wavelength switching, a dynamically changing sample may be considered virtually unchanged during a single set of shifted‐excitation in 5 ms. Slower changes in the spectrum during the total exposure will be recorded equally in all the parallelly accumulated spectra, therefore will not affect the subsequent fluorescence separation.

We note that, recently, the concept of parallelization of SERDS has also been demonstrated by Sowoidnich et al. 27, 28 independently, based on a totally different implementation from the present study. They modified the detector circuits to electrically manipulate the accumulated charges on the detector. Rejection of fluorescence and environmental background such as ambient light has been demonstrated.

## 3 MATERIALS AND METHODS

### 3.1 Experimental setup

#### 3.1.1 Optical setup

**Figure 1:**
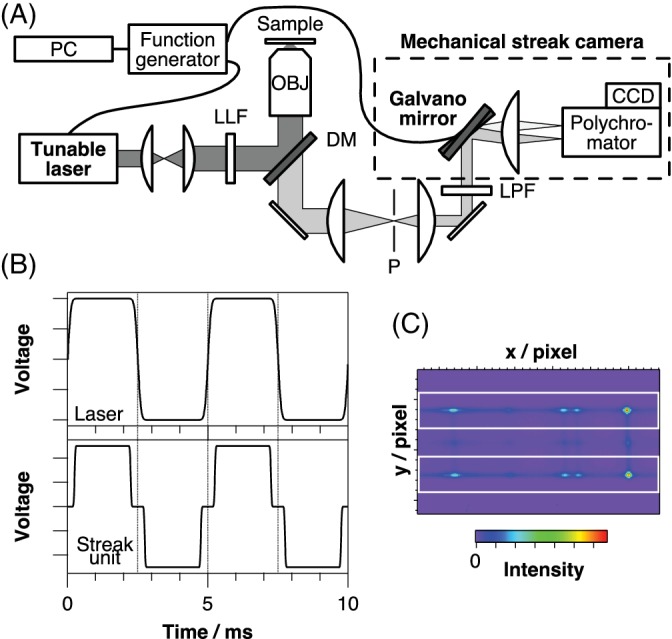
A, Schematic diagram of the experimental setup. DM, dichroic mirror; LLF, laser line filter; LPF, long‐wavelength pass filter; OBJ, objective; P, pinhole. B, Input voltage waveforms for the laser and the streak camera modulations. C, A spatial distribution of accumulated charges on charge‐coupled device (CCD) detector pixels after a single parallelized shifted‐excitation Raman difference spectroscopy (P‐SERDS) exposure. White rectangles mark the regions of interest for the detector readout

Figure 1A shows a schematic of the developed system. The light source was a CW wavelength tunable diode laser equipped with an external cavity (TOPTICA Photonics AG, DL Pro, 488 nm). First, the beam diameter of the laser output was expanded by a telescope and then introduced into an inverted microscope (Olympus, IX‐71) by a dichroic filter (LPD01‐488RU; Semrock). The incident light was focused onto a sample by an objective (40×, NA 1.3, oil immersion), and the backward‐scattered Raman signal was collected by the same objective. The back‐propagating signal was separated from the excitation laser by transmitting through the dichroic filter, and then, focused onto a confocal pinhole at the microscopic imaging plane by a second objective. After passing through the pinhole, the signal was re‐collimated and transmitted through a pair of Rayleigh rejection filters (488 LPF; Iridian Spectral Technologies), then guided into a mechanical streak camera unit for the recording of Raman spectra. The laboratory‐built streak unit was composed of a uniaxial galvano mirror (VM500plus; Cambridge Technology), a focusing lens, a polychromator (iHR‐320; HORIBA Jobin Yvon) and a charge‐coupled device (CCD) detector (Spec‐10 2 KB‐EV/LN; Princeton Instruments). The galvano mirror was placed at the back focal plane of the focusing lens to the polychromator with its scanning axis aligned to the opening of the entrance slit of the spectrometer.

#### 3.1.2 Synchronization of the laser and the mechanical streak camera

The output wavelength of the laser system and the vertical detection position of the streak unit were both controlled by external voltage signals. The laser tuning and the streak scanning were synchronized by the application of concerted voltage waveforms produced by a PC‐controlled function generator (NI USB‐6343; National Instruments), shown in Figure 1B.

Waveforms were designed so that each spectrum excited at different wavelengths was separately recorded at each designated vertical position of the CCD pixels.

Here, we note that although the DL Pro laser system was capable of mode‐hop free continuous frequency tuning in 60 GHz (0.05 nm or 2 cm^−1^) range with the help of a built‐in feed‐forward adjustment circuit, we deliberately operated the laser without the feed‐forward function to achieve repeated mode‐hopping between two discrete frequencies with wider wavelength difference. By optimizing the center voltage and the modulation amplitude of the input damped square waveform, we manage to generate mode‐hopping cycle between two wavelengths of ca. 0.1 nm (5 cm^−1^) difference in 5 ms period (200 Hz), which was appropriate for P‐SERDS experiments.

We applied the same modulation frequency of 200 Hz to the streak unit. In order to avoid spectral mixing due to timing jitter, the input waveform was cycled through three voltage levels in anomalous timing. The higher and lower voltage levels with longer duration were used to direct the signal light to regions of interest (ROIs) of the CCD readout, and the short duration intermediate level is used to discard the emission during the laser wavelength transition to outside of ROI. The typical spatial distribution of charges on the detector after a single P‐SERDS exposure is represented in Figure 1C, in which the two rectangles designate the ROIs. The vertical pixels within each region are binned before the digitization step to yield single spectral output for each ROI.

#### 3.1.3 Parallelized shifted‐excitation Raman difference spectroscopy measurement conditions

The P‐SERDS measurements of artificially prepared dye samples were performed under the synchronized modulation frequency of 200 Hz, with the excitation wavelength shift of 0.07 nm (3.0 cm^−1^). The average laser power at the sample and the exposure duration were 1.0 mW and 60 seconds, respectively. For the time‐lapse P‐SERDS measurements of algae cells, the excitation wavelength shift and the laser power were set to 0.13 nm (5.3 cm^−1^) and 2.7 mW, respectively. Other measurement parameters were the same as those for the dye samples.

### 3.2 Spectral analysis

All the data analysis, except for multivariate curve resolution‐alternate least squares (MCR‐ALS), was performed using laboratory‐developed analysis programs on Igor Pro 7 or 8 (Wavemetrics, Inc.). MCR‐ALS with the non‐negativity constraint was performed using Sci‐kit learn package (skleran.decomposition.NMF, version 0.20.3) 29 on Python.

#### 3.2.1 Correction and calibration

Prior to SERDS analysis of the acquired spectra, the obtained spectra were subject for spectral preprocessing for the correction and calibration. Spectral abscissa of each ROI is individually calibrated to wavelength by reference to atomic emission lines of a neon lamp, and Raman shift by Raman peaks of indene. The spectra were, then, individually processed for dark count subtractions, pixel sensitivity corrections by white light, and then resampled by interpolation at common wavelength series of a constant wavenumber interval (2.2 cm^−1^). Resampling was necessary because the raw spectra from different ROIs were sampled at slightly different wavelength points.

#### 3.2.2 Differential spectra

After the preprocess had been performed, each paired Raman spectra, measured simultaneously in a single exposure, were subtracted one from another and divided by the excitation frequency shift in wavenumber unit (typically 3‐5) to yield a differential Raman spectrum. A reconstructed Raman spectrum was calculated by a simple one‐dimensional numerical integration of the differential spectra; wherein, the integral constant was chosen so that the minimum value of the output spectrum became zero. Note that this choice of integration constant assumes that the observed Raman spectra contain the region of no Raman band in the observation window. This assumption may be generally valid when the “silent” region is included. However, to avoid ambiguity in the discussion, the reconstructed spectra are mainly used for the qualitative evaluation of Raman responses in this study. Alternative reconstruction algorithm based on a deconvolution calculation 20 without an apodization function was also applied to the P‐SERDS results but yielded poorer result by the massive increase in the noise level. A P‐SERDS time‐lapse spectral data set is constructed from a time‐lapse observed spectra series by converting every parallel measurement pairs to differential spectra.

#### 3.2.3 Multivariate decomposition analyses

Principal component analysis (PCA) mathematically reconstructs the input data based on its covariance to a set of principal components (PCs). Since the noise in the data has little covariance to physical signals, the process can effectively separate the signals from the noise. The number of significant (non‐noise) PCs was estimated based on a statistical test for the noise characteristics of both loading (spectral) and score (temporal) vectors 30. MCR‐ALS analysis of a denoised‐reconstructed P‐SERDS data was performed in the following procedure. First, the number of significant PCs was estimated. Then, a denoised P‐SERDS data was calculated by matrix multiplication of truncated scores and loading matrices, holding only the significant components. This denoised P‐SERDS data was transformed to reconstructed Raman data by numerical integration; then, after the negative values in the matrix were clipped to zero, the data was subjected to MCR‐ALS.

#### 3.2.4 Background subtraction

Fluorescence rejection by polynomial baseline fitting was performed using the procedure outlined in the literature 15.

### 3.3 Samples

Fluorescein (Wako Special grade) and ethanol (Guaranteed Reagent grade) were both purchased from Wako Pure Chemical Corporation and used as received. The algae cells, *Chlamydomonas* sp. JSC4, isolated from the coast of Southern Taiwan, were obtained through the courtesy of Dr. Shih‐Hsin Ho and Dr. Liang‐da Chiu. The cells were cultured in modified Bold 6 N medium 31, and then treated with 3‐day N‐depletion environmental stress condition for accumulation of lipids and carbohydrates. 32

## 4 RESULTS AND DISCUSSION

**Figure 2:**
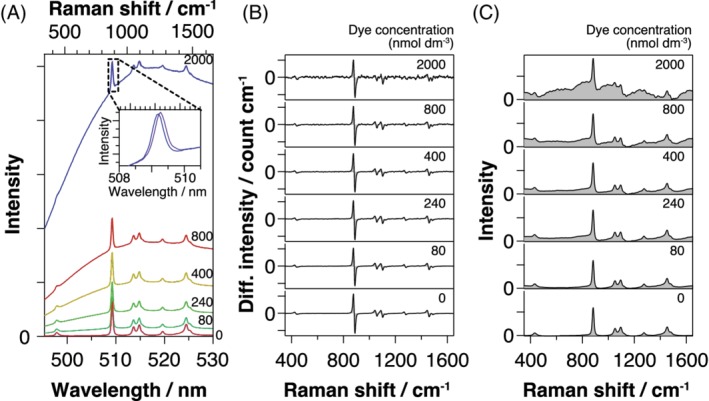
Observed Raman scattering (A), differential (B) and reconstructed (C) spectra of dilute fluorescent dye in ethanol at varying concentrations designated on the graph in nmol dm^−3^ unit. The spectra in A are plotted on a common vertical axis without any offsets. The inset is a magnified view of the dotted area. Spectra in B and C are plotted on individual vertical axes and are equally scaled. Differential intensity is abbreviated as diff. intensity

We examined the fluorescence rejection capability of our proposed method using artificially prepared samples that exhibit stationary fluorescence background. Figure 2A shows observed spectra, by the P‐SERDS method, of dilute fluorescent dye, fluorescein, in ethanol at different concentrations (0, 80, 240, 400, 800, and 2000 nmol dm^−3^). The spectra are plotted against wavelength (bottom axis) and, for the reference, the corresponding Raman shift for one of the excitation wavelengths is displayed on the top axis. Inset displays the expanded view of the graph for the 2000 nmol dm^−3^ sample, in which paired spectra with shifted vibrational bands are made visible. As is expected, each pair of spectra almost perfectly overlaps one another except for the small Raman peak shifts due to excitation wavelength shift. Every spectrum exhibits characteristic vibrational bands of ethanol at 432, 882, 1051, 1094, 1274, 1452 and 1480 cm^−3^. On top of these bands, broad fluorescence background emerges to rise concertedly with dye concentrations.

Figure 2B shows differential spectra calculated from the paired spectra in Figure 2A. All the spectra manifest substantial signals of dispersive band shape, and the spectral patterns are the same for all the samples irrespective of their dye concentrations, indicating adequate fluorescence rejection by the proposed method. For the evaluation of qualitative Raman responses, reconstructed spectra were calculated by numerical integrations of the differential spectra and are shown in Figure 2C. The reconstructed spectrum of neat ethanol sample (0 nmol dm^−3^) was identical to the original observed Raman spectrum. Hence, we conclude that no Raman vibrational information was lost by P‐SERDS analysis. In the case of fluorescent samples, slight band shape distortions with a fluctuating baseline become more apparent in the reconstructed spectra as the dye concentration increases. Nevertheless, all the reconstructed spectra present the characteristic peak pattern of ethanol and are proven to be useful for the qualitative analysis, such as general bands assignments and spectral matching. The possible origins of the band shape distortion are discussed in the latter paragraph.

**Figure 3:**
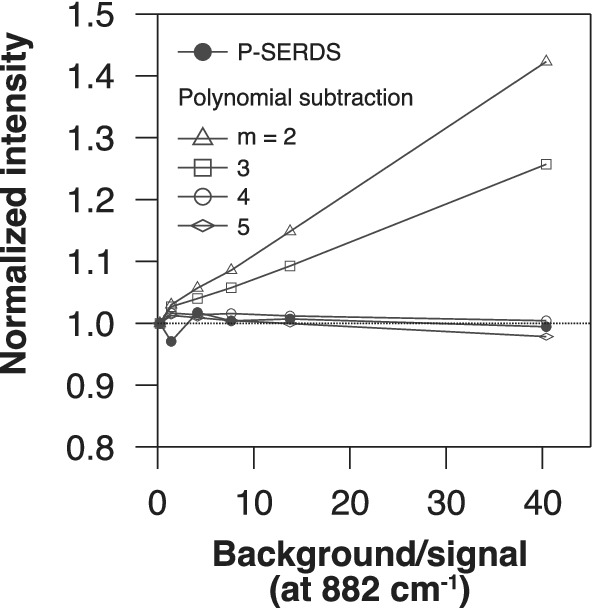
Dependence of fluorescence rejected Raman intensity on the background‐to‐signal ratio (B/S) of the original spectra. Fluorescence rejection methods are indicated in the legend. For the polynomial fitting method, *m* designates the order of polynomials. Each plot is normalized with respect to the intensity of neat solvent (B/S = 0)

In order to validate the quantitative Raman response in the differential spectra, we plotted the ethanol peak intensity at 882 cm^−1^ (estimated by the minimum to maximum intensity difference of the dispersive curve) against the background‐to‐signal ratio (B/S) of the original spectra. The result is shown in Figure 3, together with the results obtained by ad hoc background subtraction method by the baseline fitting to second‐ to fifth‐order polynomials. For the sake of comparison, each plot was normalized with respect to the peak intensity of the neat solvent at B/S = 0. We assume the ethanol concentration to be approximately constant because, for all of our samples, the dye concentrations were dilute. Therefore, the Raman response plot should ideally show the constant values of unity. Our P‐SERDS result (filled circle) exhibits almost constant unity value regardless of the background level, suggesting a robust quantification performance against interfering background.

On the other hand, the polynomial subtraction methods show highly dependent results (open symbols) on the choice of polynomial order. When polynomial order is low (2,3), the estimated Raman intensity follows linear dependence to the B/S ratio. This behavior is due to the presence of the residual background component in their Raman spectra after the background subtraction. Although the use of higher polynomial order (4,5) seems to resolve the problem, the result demonstrates one of the inherent drawbacks of the method, in which the analysis is susceptible to the choices of the analytical parameter.

Next, we applied our P‐SERDS method to living cells in order to demonstrate its feasibility to temporally unstable samples. We chose algae cells for this purpose. Microalgae are a large group of unicellular species possessing a photosynthesis system. They are considered as a prospective source of biofuel production, and much research has been devoted to engineering novel or enhanced synthesis pathways of energy‐rich metabolites, lipids and carbohydrates in algae 33. Recently, we have reported the application of Raman spectroscopy to algae characterization for a label‐free and non‐destructive evaluation of biofuel production 32. In our previous study, high fluorescence background from the photosynthesis system as well as the inhomogeneous and dynamic intracellular structures were major limiting factors which degrade the speed and accuracy of the quantification. In the present study, we applied the P‐SERDS method to individual algae cells for a fast global (average) Raman spectral acquisition by continuously and randomly moving the laser spot inside the cell during a single exposure for one minute. The measurement was consecutively repeated 30 times for the same cell to record a time‐lapse Raman spectra series of a single alga, and the whole measurement was replicated for six individual cells.

**Figure 4:**
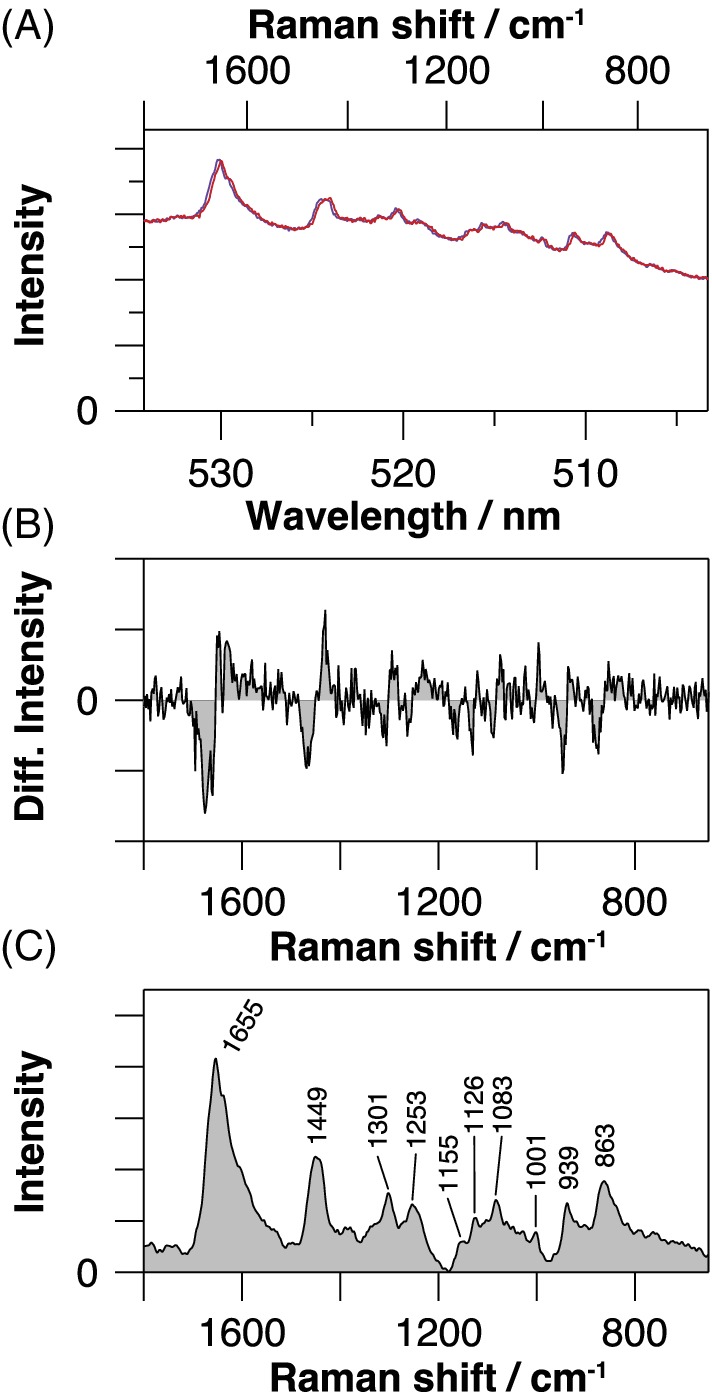
Observed Raman scattering (A), differential (B) and reconstructed (C) spectra of a single alga. The blue and red spectra in A are average Raman spectra excited by different wavelengths (487.36 and 487.23 nm, respectively) and are plotted on a common wavelength axis. The corresponding Raman shift for blue spectrum is displayed on the top axis. Differential intensity is abbreviated as diff. intensity

Time‐lapse spectra series (Figure S1) of a single algae cell observed by repeated Raman measurement for 30 minutes duration show a drastic temporal change in the background level due to fluorescence bleaching. Large intensity drops between the consecutively acquired spectra indicate that this dynamic spectral change had taken place even during each one‐minute exposure. We also anticipated the spectral change during an exposure due to the continuous laser spot movement inside the spatially inhomogeneous cell. Despite such highly dynamic measurement conditions, the P‐SERDS method successfully recorded identical baseline profiles for parallelly accumulated spectral pairs by virtue of the use of high‐frequency modulation to the excitation. Average spectra of the whole time‐lapse series from a single cell are presented in Figure 4A, together with the corresponding differential spectrum (B) and the reconstructed spectrum (C). In the differential spectrum, fluorescence was effectively canceled out, and distinct dispersive bands originating from genuine Raman signals were unequivocally observed. The reconstructed spectrum exhibited numbers of characteristic peaks of biomolecules, possibly proteins, lipids and glycogens, readily usable for further qualitative and quantitative analysis. Possible assignments of some of the prominent bands are as follows: proteins (amide I at around 1650 cm^−1^, CH_3_ deformation [deform.] at 1449 cm^−1^, amide III at 1253 cm^−1^, phenylalanine ring at 1001 cm^−1^); lipids (C=C stretch [str.] at 1655 cm^−1^, CH_2_ scissors at 1449 cm^−1^, CH_2_ twist at 1301 cm^−1^, =C—H bend at 1253 cm^−1^, skeletal C—C str. at 1126 and 1083 cm^−1^) and glycogens (C—O—H bend at 939 cm^−1^, C—O and C—C str. and C—H bend at 1155, 1083 and 863 cm^−1^) 34, 35, 36.

**Figure 5:**
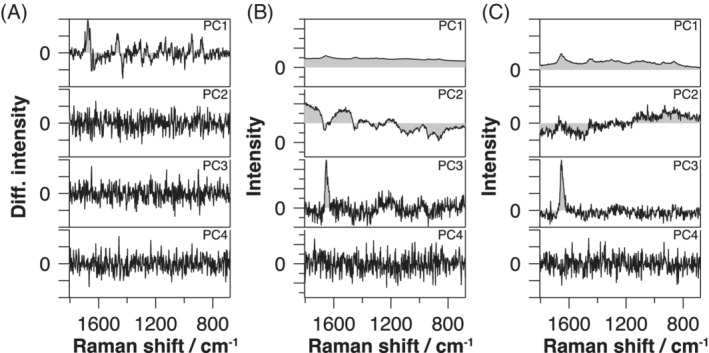
First four loading spectra of principal component analysis for the parallelized shifted‐excitation Raman difference spectroscopy (P‐SERDS) (A), the original (B) and the third‐order polynomial subtracted (C) time‐lapse data sets, obtained from a single cell. Spectra are plotted on individual vertical axes and are equally scaled within each column. Diff. intensity, differential intensity; PC, principal components

Next, to assess the efficacy of fluorescence rejection in multivariate analysis, we constructed a P‐SERDS time‐lapse spectral data set, and it was subjected to PCA. We also performed the polynomial baseline subtraction to the same time‐lapse series. This polynomial subtracted data set and the original fluorescence unremoved time‐lapse series were also processed by PCA for comparison. Table 1 summarizes the number of significant components identified for each data set. Loading spectra of the first four PCs of the P‐SERDS, the original and the polynomial subtracted data sets are represented in Figure 5A‐C, respectively.

**Table 1:** Number of significant components derived by principal component analysis

Fluorescence rejection method			*m*th‐order polynomial baseline fitting			
Data set	P‐SERDS	Unrejected (observed)	*m* = 2	*m* = 3	*m* = 4	*m* = 5
One cell	1	3	3	3	4	4
Six cells	2	5	5	5	7	7

PCA analysis revealed that the original observed time‐lapse data set of a single alga has a little complicated nature than expected, carrying three independently varying components (Figure 5B). In general, direct interpretations of individual PCs are not straightforward because PCs merely represent statistical characteristics of the data set, and their relationship to chemical species or biological structures are indirect. Physically relevant components often appear as mixtures in the PCs. In our case, we could only speculate the physical nature of two of the variations, one as a temporary decreasing (bleaching) fluorescence and the other as an (almost) stationary Raman signal of the cell. The presence of the third component suggests that there is additional variation in the data set, which may either be a second fluorescence component with different bleaching kinetics or a second Raman component with non‐stationary kinetics. However, due to the mixing of components in the PCs, further analysis based only on the PCA result was hampered. This situation illustrates the limitation of multivariate techniques, where the presence of unwanted additional components complicates the analysis.

In contrast to the fluorescence unremoved result, the PCA result of the P‐SERDS data set exhibited benefits of prior fluorescence rejection by much simplified composition as it yielded only one significant component (Figure 5A). This sole component resembles the differential spectrum in Figure 4B, except for the sign which was determined arbitrarily, suggesting that the fluorescence contribution is effectively minimized to below noise level by P‐SERDS calculation and that a single Raman spectral pattern had been recorded during the time‐lapse series throughout. From the decrease of the number of significant components from original (3) to P‐SERDS data (1), we inferred that the diminished two varying components were two fluorescence spectra with different kinetics.

Unlike the P‐SERDS result, the polynomial fitting approach (Figure 5C) did not resolve the issue. The number of significant components did not change after the treatment by low‐order polynomial, or, even worse, increased when higher‐order polynomials were employed (Table 1). The result is most likely due to the inaccuracy of the baseline estimation by the fitting. The PCA exposed the residual fluorescence and artifactual components introduced from over or under subtraction of the baseline.

Next, we further extended our study to demonstrate the P‐SERDS application to assess cell‐to‐cell individuality of algae by multivariate resolution techniques. A time‐lapse Raman spectra series of six individual algae cells were concatenated to form a large matrix. This total data set was then processed for PCA analysis. As a result, we found two significant components (Figure S2). The presence of multiple Raman components in PCA is an indication of the cell‐to‐cell variation of their biomolecular composition ratio. In order to determine the relevant biomolecular species, we employed MCR‐ALS method with the non‐negativity constraint 11 on a denoised‐reconstructed P‐SERDS data set that had derived from the PCA result. MCR‐ALS is an alternative method to PCA in finding bilinear matrix decomposition of the input data matrix. By the use of non‐negativity constraint, which reflects the physical nature of Raman scattering, the method tends to find a chemically interpretable decomposition solution 37. The user must supply the number of components, and in the present case, we designated two components based on the PCA result.

**Figure 6:**
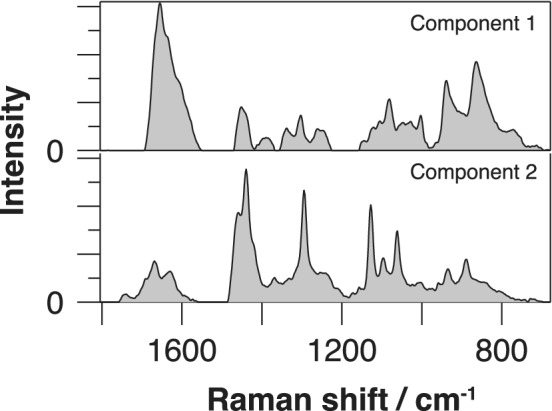
Decomposed Raman spectra derived from multivariate curve resolution‐alternate least squares of parallelized shifted‐excitation Raman difference spectroscopy (P‐SERDS) reconstructed data set

The decomposed Raman spectra obtained from MCR‐ALS analysis, presented in Figure 6, displays characteristic peaks of proteins and glycogens (top) and saturated lipids (bottom), with small spectral crosstalks. The lipid spectrum exhibited single sharp peak at 1295 cm^−1^, as well as triplets at 1128, 1099 and 1061 cm^−1^, which are the signature of all‐trans C—C chain conformation of saturated fats in solid phase 35, suggesting the presence of highly condensed lipid package in some of the cells. The result suggests a diversity of lipid ratio to other biomolecules among the cell population, which may indicate the individuality of lipid production and storage ability. We note that PCA analyses to the original observed and the polynomial subtracted data sets yield larger numbers of significant components (Table 1) due to the fluorescence interference. Because of the complication raised by an increased number of components, further MCR‐ALS analyses failed to generate clear resolution of the varying biomolecular species as was possible by the P‐SERDS data.

Next, we remark the applicability of the P‐SERDS method to various Raman measurements. The method can also be used to reject non‐fluorescent background, for example, spontaneous emissions from the sample and ambient light from the environment, as long as they are independent of the excitation wavelength shift. The essential devices, the tunable laser and the galvano mirror unit, can be easily incorporated into a working single‐focus Raman spectroscopic system equipped with a two‐dimensional detector, with the least modification to the optical layout. Therefore, the method is not limited to a biological investigation and applies to a wide variety of research subjects whenever the interference of background is inevitable.

Finally, we discuss some of the limitations of the present P‐SERDS method. Firstly, as is partly seen in Figure 3, the P‐SERDS also suffers from the common drawback of difference spectroscopy, in which the signal‐to‐noise ratio is generally poor as it focuses specifically on the variations in the signal, not on the signal amplitude itself. In the case of a static background, if reliable background spectra can be obtained by, for example, separate measurement, simple background subtraction analysis may deliver better results. Second, since the spectra accumulated in parallel propagate along different optical paths after the streak camera, asymmetry in the optical configuration due possibly to misalignment and aberrations would result in the additional difference in the observed spectra. This additional difference may be minimized by careful corrections and calibrations of the data, but it may not be completely canceled out in the subtraction step. Practically, the error caused by this artificial difference is negligibly small in the differential P‐SERDS spectra, but it may be exaggerated in the reconstructed spectra to produce distorted Raman band shapes and fluctuating baseline, and, therefore, could affect the qualitative analysis. We noticed that the aberration in the imaging spectrometer was the primary source of the error in our setup. Restricting the observation ROIs to the central part of the imaging plane, where the aberration is at most corrected, helped to reduce the distortion. Alternatively, the lock‐in CCD‐based SERDS technique recently proposed 28 circumvent the aberration issues at the expense of reduced effective CCD pixel (row) number.

## 5 CONCLUSION

In this study, we have developed P‐SERDS by the synchronized operation of a mechanical streak camera with a wavelength tunable light source. The parallel detection scheme proposed by the present method facilitates the effective and accurate fluorescence rejection from the temporary varying system with much faster dynamics than the spectral acquisition rate, thus expanded the scope of SERDS technique to include broader and more practical biological application. The highly accurate background rejection performance of P‐SERDS for such dynamic samples is demonstrated to be beneficial, especially for the multivariate analysis of Raman data set by reduction of the number of retained compositions. This simplification of the analytical result not only helps in further quantitative and qualitative assessments but also promotes the development of clearer biochemical insight into the target system. We believe that the present method has enormous potential in boosting the reliability and applicability of Raman spectroscopy in biological research, and thus empowers related discrimination, diagnosis and quantification applications in the near future.

## CONFLICT OF INTEREST

The authors declare no financial or commercial conflict of interest.

## Supporting information


**Figure S1.** Time‐lapse Raman spectra series of a single alga by parallelized shifted‐excitation Raman difference spectroscopy (P‐SERDS).
**Figure S2.** First five loading spectra of principal component analysis for the concatenated time‐lapse series of six algae cells.
