# Supplementary material for: Parallelized shifted‐excitation Raman difference spectroscopy for fluorescence rejection in a temporary varying system
Source: J Biophotonics. 2019 Aug 28;12(12):e201960028. doi: 10.1002/jbio.201960028 (PMC7065630; doi:10.1002/jbio.201960028)
Supplement: Supplementary file 1 — Figure S1. Time‐lapse Raman spectra series of a single alga by parallelized shifted‐excitation Raman difference spectroscopy (P‐SERDS). Figure S2. First five loading spectra of principal component analysis for the concatenated time‐lapse series of six algae cells. [file JBIO-12-e201960028-s001.docx]

Supporting Information

Title: Parallelized shifted-excitation Raman difference spectroscopy for fluorescence rejection in a temporary varying system

Rintaro Shimada^*^, Takashi Nakamura, and Takeaki Ozawa^*^


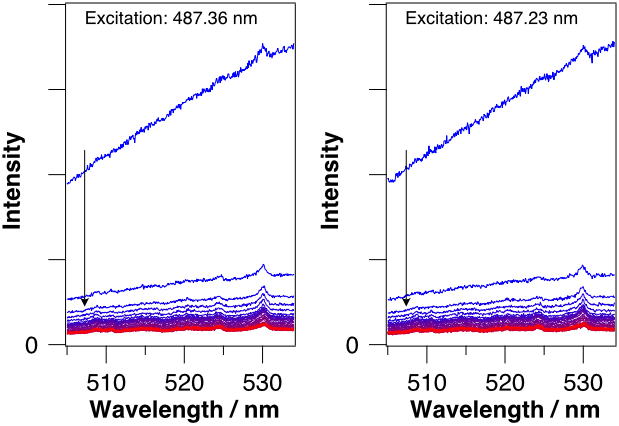


**FIGURE S1.** Time-lapse Raman spectra series of a single alga by P-SERDS. The two graphs correspond to the spectra excited by different wavelengths, of which exact values are annotated. The simultaneously recorded spectra pairs among two graphs are coded by the same color. The arrows in the graphs indicate the direction of spectral change during the time-lapse series.

**
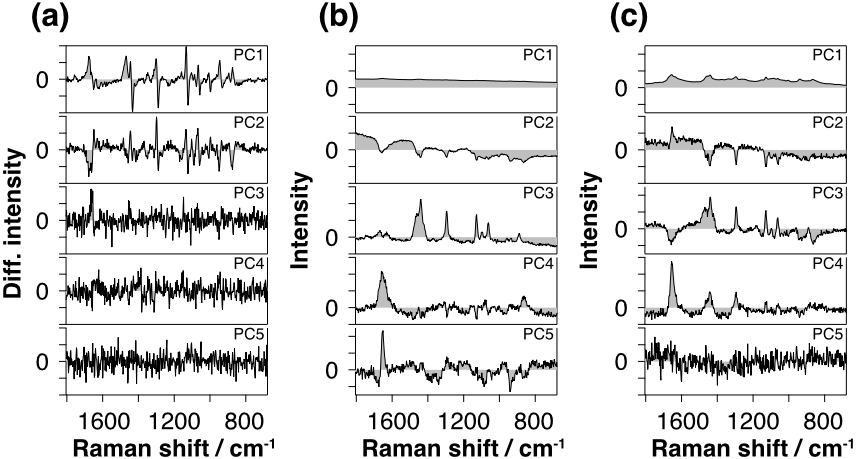
**

**FIGURE S2.** First five loading spectra of principal component analysis (PCA) for the P-SERDS (a), the original (b), and the third order polynomial subtracted (c) time-lapse data sets, obtained from the combined data set of six algae cells. Spectra are plotted on individual vertical axes and are equally scaled within each column. Diff. intensity, differential intensity; PC, principal components.
